# Pregnancy Activity Levels and Impediments in the Era of COVID-19 Based on the Health Belief Model: A Cross-Sectional Study

**DOI:** 10.3390/ijerph19063283

**Published:** 2022-03-10

**Authors:** Hongli Yu, Juan He, Anna Szumilewicz

**Affiliations:** Department of Sport, Gdansk University of Physical Education and Sport, 80-336 Gdansk, Poland; juan.he@awf.gda.pl (J.H.); anna.szumilewicz@awf.gda.pl (A.S.)

**Keywords:** pregnant women, health belief level, prenatal physical activity, individual perception, health-belief model

## Abstract

Physical activity (PA) and exercise benefit both the mother and the fetus. Many pregnant women avoid or severely limit PA, leading to complications before and after delivery. This study elucidated the precise effect of each moderator variable on prenatal physical activity (PPA) by examining demographic factors, the PPA-related health belief level (HBL), and the current PPA level. The health belief model (HBM) in conjunction with the international prenatal physical activity questionnaire was used. The HBL in pregnant parous women (PPW) (3.42) was significantly higher than that in nonpregnant nulliparous women (NNW) (3.06). The PPA level in pregnant nulliparous women (PNW) (5.67 metabolic equivalent-hours per week (MET-h/week)) was lower than in the PPW (6.01 MET-h/week). All HBM dimensions (except for perceived barriers) were positively correlated with exercise expenditure in both PNW and PPW. According to the regression tree, participants in PNW aged ≤ 23 years with annual household incomes > CNY 100,001–150,000 had the highest energy expenditure (10.75 MET-h/week), whereas participants in PPW with a perceived benefit score of >4 had the highest energy expenditure (10 MET-h/week). The results demonstrated that the HBL in all groups was acceptable, whereas the PPA level was lower than the recommended PA level. In both PPW and PNW, the HBL was most strongly correlated with exercise expenditure. There is an urgent need to organize public-interest courses to alleviate household expenditure, raise the HBL about PPA in pregnant and NNW, and ensure personal health in the context of COVID-19.

## 1. Introduction

The World Women’s Organization (WWO) was established to defend women’s rights in 1947 [1], and the 2030 agenda for sustainable development goal (SDG) 5 points to achieving gender equality and empowering all women and girls, which indicates that women have gained global attention [2]. Pregnancy represents a unique period in a woman’s life, and women’s health has garnered much attention in the context of women’s rights advocacy. Although having children has numerous benefits, women can also face many challenges due to pregnancy, such as obesity, diabetes, nausea (with or without vomiting), discomfort in the pelvic girdle, and other musculoskeletal issues [3].

Studies have demonstrated that being active and leading a healthy lifestyle while pregnant can reduce both the mother’s and baby’s risk of acquiring chronic diseases [4]. The maternal advantages of exercise include increased fitness; avoidance of excessive pregnancy weight gain, weight retention, and possibly obesity, gestational diabetes, hypertension, and maternal depression [5]; and a decrease in cesarean-section rates [4]. Maternal exercise is also related to healthy birth weight and the prevention of chronic illness in children [6]. Recent studies have shown that maternal physical activity (PA) plays a positive role in the modulation of the progeny’s phenotype, giving the offspring improved health potential [3]. The available data unequivocally demonstrate that PA and exercise should be components of a healthy lifestyle for pregnant women. Unfortunately, many pregnant women forego or severely restrict their PA and exercise [6]. The terms of PA and exercise are clarified in this study to understand them correctly. PA is defined as any bodily movement produced by the contraction of skeletal muscles that results in a substantial increase in caloric requirements over resting energy expenditure [7,8]. Exercise is a physical activity consisting of planned, organized, and repeated body movement to enhance and maintain one or more components of physical fitness [7] and extend life [9]. Exercise is thus a subcategory of physical activity.

Pregnancy is a memorable life stage that may elicit distinct variables that motivate and impede PA [10]. Pregnant women may want to perform PA for their own health and that of their baby, but they may struggle to do so. Recent studies have revealed that pregnant women tend to be sedentary [10]. Major reasons and factors for this include (1) a lack of desire to be active, which pregnant women often mention as a barrier to exercise [11]; (2) unawareness of the value of prenatal physical activity (PPA), how much exercise is needed, and how to exercise safely [10]; (3) social variables, such as a lack of support from friends, family, and doctors to be active, which can significantly influence PPA levels; and (4) traditional culture, as pregnancy has historically been viewed as a time for relaxation and recuperation [12]. However, these studies have not proposed specific strategies for solving these problems, and the listed reasons and factors may vary by the location, culture, and limitations of the coronavirus disease 2019 (COVID-19)-pandemic era. Health status and geographic location are crucial variables that directly and indirectly impact PPA behavior through other determinants [13]. This is particularly true in China, where the two-child policy is liberalized. As the “two-child” fertility rate rises, barriers may shift as an outcome of this policy [14]. Meanwhile, COVID-19 and its corresponding isolation periods may harm the health of pregnant women and introduce various PPA dilemmas. 

Consequently, it is essential to study the variables and obstacles influencing PA among pregnant women in China and explore effective strategies, considering differences in geography, culture, and the prevalence of COVID-19. This study can contribute to health and well-being, equity, quality education, and society safety under the international spotlight [15]. Moreover, it falls within the scope of the UN’s Sustainable Development Agenda 2030. Previous studies found that Chinese women were less active when pregnant than they were before they became pregnant [16] and that exercise intensity and duration also reduced [17]. However, the following factors are unclear: (1) the level of PPA during the COVID-19 pandemic; (2) how psychological, demographic, and social factors synthetically influence PPA; (3) which of these variables are the most prominent in Chinese pregnant women during the COVID-19 era; (4) the cognitive levels of PPA in nonpregnant nulliparous women who may also experience pregnancy complications in the future.

The health belief model (HBM) [18] is a classic and widely used psychological theory in health science that may be employed to determine the most critical factors across geographies, economies, and cultures and shed light on the relationship between psychological, demographic, and social factors and PPA. The HBM defines the key factors that influence health behaviors as an individual’s perceived threat to sickness or disease (perceived susceptibility), belief of adverse consequence (perceived severity), potential positive benefits of action (perceived benefits), perceived barriers to action, exposure to factors that prompt action (cues to action), health motivation, and confidence in ability to succeed (self-efficacy) [18]. The HBM provides a simple method of understanding factors that influence behavior and identifying specific behavior-change techniques that can affect these factors to increase the likelihood that the desired behavior will be enacted [19]. The HBM is an effective tool, which has been successfully applied in health education and health promotion for explaining and promoting preventive health behaviors [19]. Using the HBM, researchers may discover likely methods and resources to improve women’s desire and capacity to engage in or maintain PA throughout pregnancy during the COVID-19 pandemic.

Consequently, using the HBM in conjunction with the international prenatal physical activity questionnaire (PPAQ) [20], the current research focused on the following objectives: (1) to determine the health belief level (HBL) and PA among Chinese women who are nonpregnant nulliparous, pregnant nulliparous, and pregnant parous; (2) to examine the demographic factors and HBM dimensions associated with the current PPA; (3) to predict the values of PPA, which may be useful in establishing population subgroups in need of intervention in order to determine the best route for increasing the PPA level during COVID-19. On the basis of the above information, we hypothesized that (1) the PPA level would be lower than the recommended PA level during COVID-19; (2) nonpregnant nulliparous women would have lower HBLs than pregnant nulliparous and pregnant parous women; and (3) demographic factors and HBL are correlated with PPA.

## 2. Materials and Methods

### 2.1. Study Design and Respondents

It was a cross-sectional study in a convenience sample of nonpregnant nulliparous, pregnant nulliparous, and pregnant parous Chinese women. They were asked to voluntarily participate in the research through an e-questionnaire displayed at five geographically distributed medical practices across several regions in China: Sichuan, Hubei, Shanxi, Chongqing, and Guangdong. Demographic factors, HBM dimensions (perceived susceptibility, perceived severity, health motivation, perceived benefits, perceived barriers, self-efficacy, and cues to action), and PPA levels (only in pregnant nulliparous and pregnant parous women due to the specificity of the PPAQ) were collected in the survey. Prepregnancy PA was not collected from the pregnant participants; consequently, the PA of nonpregnant nulliparous women was not investigated. The inclusion criteria for nonpregnant nulliparous respondents were as follows: 20-to-50-year-old healthy females without contraindications to exercise, nonpregnant nulliparous women, and women with no mental or cognitive problems. For pregnant respondents, the inclusion criteria were as follows: 20 to 50 years old, single pregnancy without contraindications to exercise, no history of recurrent miscarriage or preterm delivery, no early membrane rupture or vaginal hemorrhage, no placenta previa, no severe anemia or other systemic illnesses, and no mental or cognitive problems. The exclusion criteria were as follows: age under 20 years old or over 50 years old, multiple previous pregnancies, current pregnancy with obstetrical and medical illnesses necessitating PA restriction, inability to complete the questionnaire, and refusal to participate in the research. All procedures performed in the study involving human participants were in accordance with the Bioethics Commission at the District Medical Chamber in Gdansk (KB—8/21 and 8/21a). All participants provided written informed consent.

### 2.2. Materials

#### 2.2.1. Survey Tool

Following the HBM, the researchers created the questionnaire. The survey instrument was created following a thorough examination of both the existing research literature and previously produced questionnaires evaluating pregnant women’s demographic factors and HBM dimensions. The PPAQ was also used to determine the pregnant respondents’ PA levels. The survey was subsequently pilot tested to assess its content validity and clarity by medical practitioners, academic researchers, and postgraduate students (*n* = 10) who also provided feedback that contributed to the final version of the instrument. The survey was divided into three parts: participants’ demographic features, PPA-related health beliefs, and PPA during pregnancy.

The following were the exact contents of the survey. (1) Demographic variables were collected and evaluated, including the participant’s age, combined family income, marital status, education level, job status, and number of children. (2) The PPAQ was created in the United States and is now widely utilized globally. The Chinese version was introduced and translated by Zhang Yan et al. with a 0.940 content validity and 0.944 retest reliability (*p* < 0.01) [20]. This version of the PPAQ contains four domains: housework (14 items), transportation (4 items), occupational activities (8 items), and exercise (5 items). The PPA value in this research was determined using the following energy-expenditure formula: metabolic equivalent (MET)-hours per week = MET coefficient of activity × duration (hours per session) × frequency (times per week) [21]. According to the recommendations of the American College of Obstetricians and Gynecologists (ACOG) regarding PA during pregnancy and postpartum [22], the PPA of 7.5 MET-h/week was deemed a sufficient PPA level in this study. A PPA of less than 7.5 MET-h per week was deemed insufficient [22]. (3) PPA-related HBL was examined using this part of the questionnaire, which included seven dimensions with 27 items according to the HBM (see Table 1 for the specific dimensions and items). The respondents assessed each item on a 5-point Likert scale, in which 5 indicated “strong agreement”; 4 indicated “moderate agreement”; 3 indicated “not sure”; 2 indicated “moderate disagreement”; and 1 indicated “strong disagreement”. Scores < 3 represented a poor HBL, scores ≥ 3 and ≤4 represented an acceptable HBL, and scores > 4 represented a good HBL. The item related to perceived barriers adopted the reverse-scoring method (i.e., higher scores indicated that fewer obstacles were encountered.). Since the number of items in each dimension varied, the mean score of each dimension (i.e., the score of each dimension/number of items) was computed for ease of comparison, and the exploratory factor analysis (EFA)-weighted score method was used to calculate the weighted HBL scores, with a higher score representing a higher PPA health belief [23].

#### 2.2.2. Pregnancy Physical Activity-Related Health Belief Model Assessment

**Figure 1:**
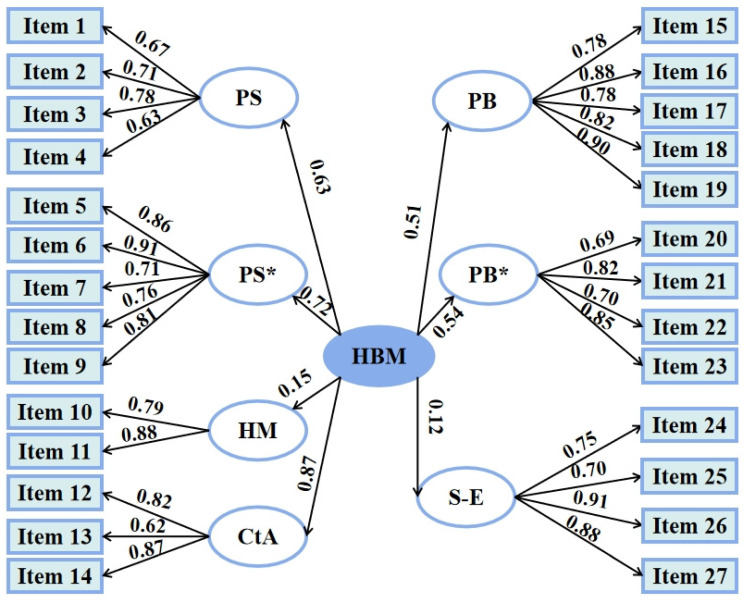
Health belief model (HBM) path diagram and the standardized path coefficient. PS: perceived susceptibility; PS*: perceived severity; HM: health motivation; PB: perceived benefits; PB*: perceived barriers; S-E: self-efficacy; CtA: cues to action; HBM: health belief model.

The reliability score of the entire HBM scale, represented by Cronbach’s alpha, was 0.91. Confirmatory factor analysis (CFA) was performed to identify the construct validity, convergent validity, and discriminant validity between factors and measurement items [24]. The standard estimate of 27 items was greater than 0.6, indicating an effective measurement relationship (Figure 1). The average variance extraction (AVE) values of the seven factors were all greater than 0.5, and the combination reliability (CR) values were all greater than 0.7, signifying that the data in this analysis had sound construct validity. The AVE square-root values of the seven factors were all greater than the absolute value of the correlation coefficient between the factors, meaning that the factors had high discriminant validity. A construct validity test of CFA was performed, and the following fit statistics were obtained: root mean square error of approximation = 0.08 (<0.1 indicates good fit), goodness of fit index = 0.91 (>0.90 indicates good fit), Chi-square value/degree of freedom (χ2/df) = 2.9 (<3 indicates good fit), and comparative fit index = 0.92 (>0.90 indicates good fit). Figure 1 presents the factor structure of the HBM and the standardized path coefficient.

#### 2.2.3. Data Collection

Data were collected using professional online questionnaire survey technology (WenJuanXin), and a link or quick-response (QR) code for the electronic questionnaire was created to make it easier for participants to scan the code and complete it on their smartphone. Data were gathered from May to August 2021. Senior midwives observed the data collection process for quality control with the express permission of participants who fulfilled the inclusion criteria. Participants were instructed on how to complete the questionnaire and were informed of issues to be addressed. Logic-based questions and limitations were added to the questionnaire survey software to ensure respondents did not miss questions and repeat engagements. Once the questionnaire was completed, the same phone could not scan the QR code. However, if the anomalous data varied considerably from the typical value, it was removed, or if the respondent’s unit of height, weight, or age was not in accordance with the requirements, it could be manually changed. According to the CFA suggestion and calculation result of sample size (α = 0.05, d = 0.3, and 1-β = 0.8) [24], 300 questionnaires were estimated to be collected. A total of 425 electronic questionnaires were collected, with 11 marked anomalous data. There were 414 valid electronic questionnaires after deleting 11 samples that departed from the normal value.

### 2.3. Statistical Methods

G*power (version 3.1.9.4) was used to compute the required sample size. Statistical Product and Service Solutions (SPSS) version 26.0 was used to analyze the validity and reliability of the health belief questionnaire. OriginPro 2021 (version 9.8.0.200, OriginLab Corporation) was utilized to conduct the analysis of variance (ANOVA) and the Pearson correlation coefficient assessment. ANOVA was chosen to evaluate variations in each group’s health beliefs and PPA levels. CFA was used to identify the construct validity, convergent validity, and discriminant validity of the factors. The Pearson correlation coefficient was performed to analyze the link between participants’ demographic features, their PPA-related HBL, and PPA. The R programming language (version R x64 4.1.1, R Development Core Team) was used to build the classification and regression tree (CART) [25] to elucidate the precise effect of each moderator variable on PA behavior in pregnant women. The binary tree serves as a logical framework for constructing prediction criteria on the basis of current research data. PPA-related factors served as the study’s input variables, with PPA energy expenditure values serving as its outcome variables.

## 3. Results

### 3.1. Demographic Characteristics

The demographic characteristics of the 414 respondents are presented in Table 2. Participants were Chinese citizens (100%); 202 (48.9%) aged 26 to 34; 259 (62.5%) were of Han nationality; 141 (34.1%) were living in urban areas; pregnant nulliparous women (42.6%) and pregnant parous women (57.4%), of whom 34.2% were in the third trimester. A total of 41.1% of the participants in this study had children. Participants with bachelor’s degrees composed more than half of the study population (50.3%), and most of their spouses also had bachelor’s degrees (46.6%). Approximately 26% of the population had an annual income below CNY 50,000 (Table 2).

### 3.2. Health Belief Level (HBL) and Prenatal Physical Activity

**Figure 2:**
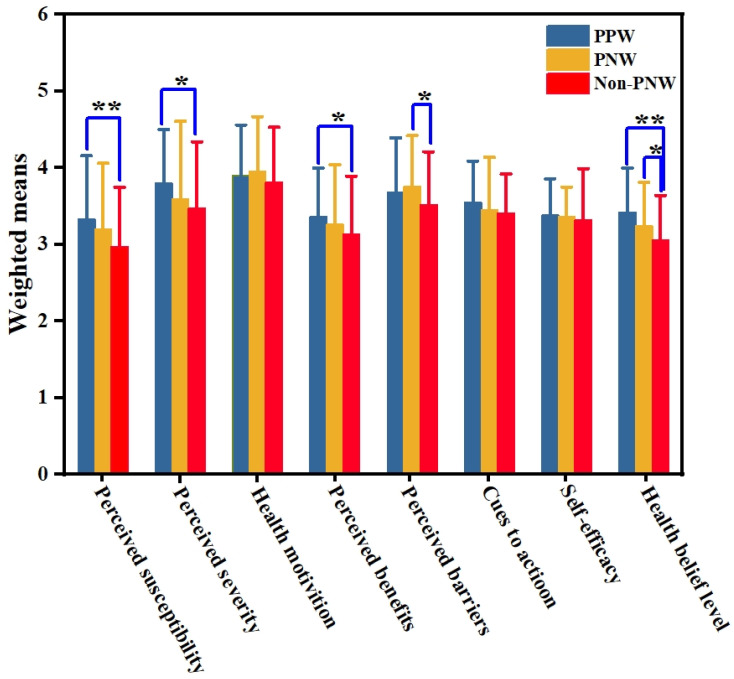
Comparison of health belief dimensions among nonpregnant, pregnant nulliparous, and pregnant parous women. Note: PPW: pregnant parous women; PNW: pregnant nulliparous women; Non-PNW: nonpregnant nulliparous women; ** *p* < 0.01; * *p* < 0.05.

Figure 2 displays the results and compares each HBM dimension and HBL among nonpregnant nulliparous, pregnant nulliparous, and parous women, as indicated by each dimension’s weighted HBL scores and mean scores. All dimensions of individual perceptions were examined, and a statistically significant difference was observed in the reported perceived susceptibility, severity, and benefits between nonpregnant nulliparous and pregnant parous women (*p* < 0.05). Nonpregnant nulliparous and pregnant nulliparous participants differed significantly in perceived barriers and HBL (*p* < 0.05), whereas health motivation, cues to action, and self-efficacy were not significantly different (*p* > 0.05). The pregnant parous group had the highest HBL (3.42 ± 0.58), followed by the pregnant nulliparous (3.24 ± 0.57) and nonpregnant nulliparous women (3.06 ± 0.58). The highest score was achieved for health motivation (3.95 ± 0.72) in pregnant nulliparous women, whereas the lowest score was achieved for perceived susceptibility (2.97 ± 0.78) in nonpregnant nulliparous women.

### 3.3. Physical Activity Expenditure during Pregnancy

**Figure 3:**
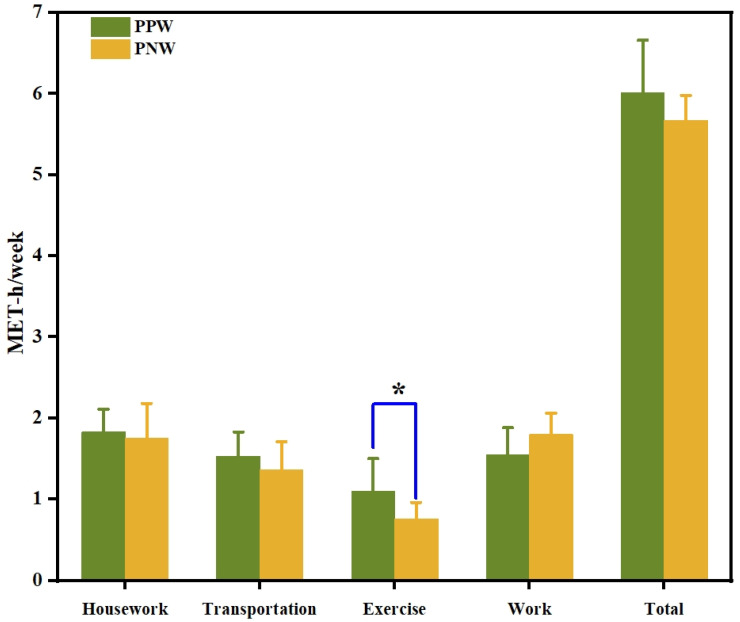
Mean difference in physical activity expenditure between pregnant nulliparous and pregnant parous women. Note: MET-h/week: metabolic equivalent-hours per week; PPW: pregnant parous women; PNW: pregnant nulliparous women; * *p* < 0.05.

Pregnant parous women had the highest energy expenditure for housekeeping (1.83 MET-h/week) and the lowest for exercise (1.10 MET-h/week), and their total energy expenditure was 6.01 MET-h/week. The pregnant nulliparous group had the highest energy expenditure for work (1.80 MET-h/week) and the lowest for exercise (0.76 MET-h/week), and their total energy expenditure was lower than that of the pregnant parous group (5.67 MET-h/week). However, pregnant nulliparous and pregnant parous women exhibited no statistically significant difference in energy expenditure for housework, transportation (driving to and from work), and work (*p* > 0.05). A significant difference in energy expenditure for exercise was observed between pregnant nulliparous and pregnant parous women (*p* < 0.05) (Figure 3).

### 3.4. The Association between Demographic Factors, Health Belief Model Dimensions, and Pregnancy Physical Activity

**Figure 4:**
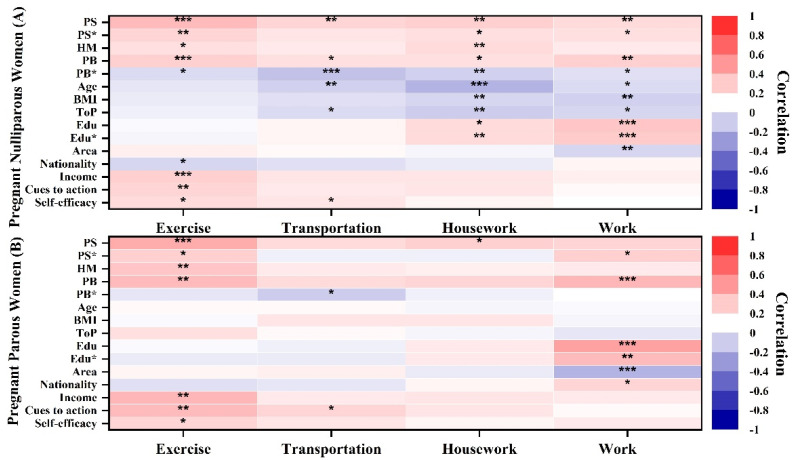
Heat maps of the Pearson correlation of demographic and health belief model (HBM) dimensions and prenatal physical activity. The pregnant nulliparous group is shown in the first map, and the pregnant parous group is shown in the second map. PS: perceived susceptibility; PS*: perceived severity; HM: health motivation; PB: perceived benefits; PB*: perceived barriers; BMI: body mass index; ToP: trimester of pregnancy; Edu: participant’s education background; Edu*: spouse’s education background; ***: significant correlation at *p* < 0.001; **: significant correlation at *p* < 0.01; *: significant correlation at *p* < 0.05. A darker color indicates a stronger association and vice versa; red is positively correlated, whereas blue is negatively correlated.

In this study, all variables were selected and classified based on the HBM categorization, and they were divided into demographic characteristics (Table 2) and HBM dimensions (Table 1). A Pearson correlation coefficient analysis was performed to assess the correlations between demographic characteristics, HBM dimensions, and PPA. All HBM dimensions were positively correlated with exercise expenditure in both the pregnant nulliparous and pregnant parous groups, except for perceived barriers. By contrast, perceived barriers, age, body mass index (BMI), and trimester of pregnancy were negatively correlated with housework and work activities in pregnant nulliparous women. Location was negatively correlated with work in both pregnant nulliparous and pregnant parous women (Figure 4).

**Figure 5:**
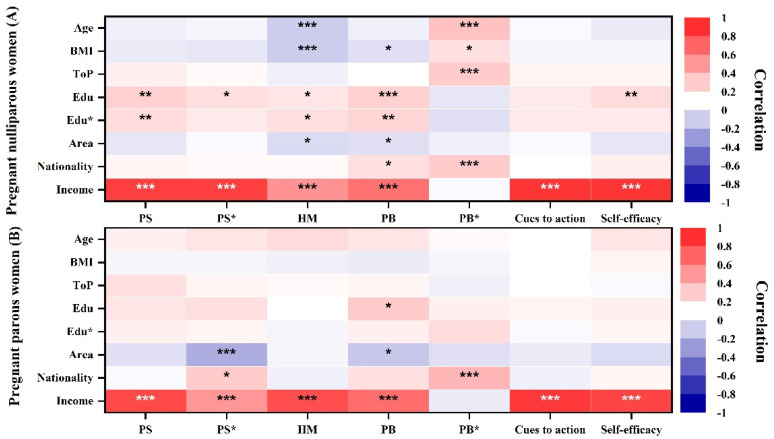
Heat maps of the Pearson correlation of demographic and health belief model (HBM) dimensions. The pregnant nulliparous group is presented in the first map. The pregnant parous group is shown in the second map. PS: perceived susceptibility; PS*: perceived severity; HM: health motivation; PB: perceived benefits; PB*: perceived barriers; Edu: participant’s education background; Edu*: spouse’s education background; BMI: body mass index; ToP: trimester of pregnancy. ***: significant correlation at *p* < 0.001; **: significant correlation at *p* < 0.01; *: significant correlation at *p* < 0.05. A darker color indicates a stronger correlation and vice versa; red is positively associated, whereas blue is negatively associated.

A Pearson correlation coefficient analysis was performed between demographic factors and HBM dimensions, taking into account the variables’ interconnectedness. Figure 5 displays the correlation between demographic factors and HBM dimensions. In both pregnant nulliparous and pregnant parous women, income was positively linked to HBM dimensions (except for perceived barriers). In the pregnant nulliparous group, the spouse’s education background was positively correlated with perceived susceptibility, benefits, and health motivation. However, in both pregnant nulliparous and pregnant parous women, the residential zone was negatively correlated with perceived benefits, and age and BMI were negatively correlated with health motivation but positively correlated with perceived barriers.

### 3.5. Construction of Classification and Regression Tree (CART)

**Figure 6:**
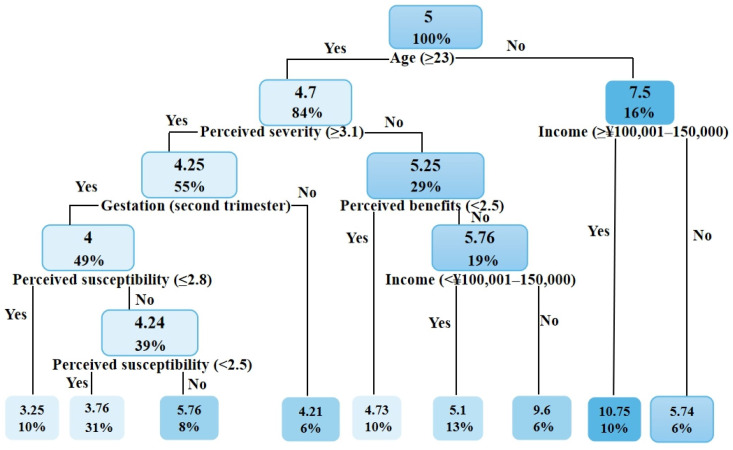
The classification and regression tree illustrated the predicted values of physical activity level in pregnant nulliparous women. Note: The values in the rectangle indicate the amount of energy expenditure (metabolic equivalent (MET)-hours per week) and the percentage of the samples taken.

**Figure 7:**
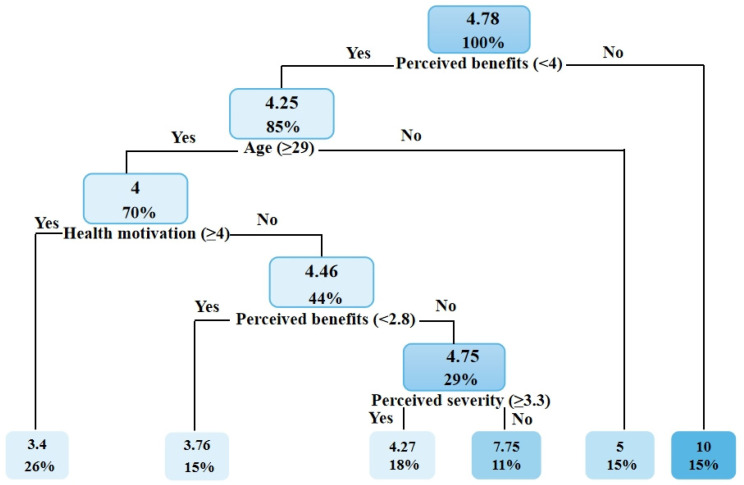
The classification and regression tree illustrated the predicted values of physical activity level in pregnant parous women. Note: The values in the rectangle indicate the amount of energy expenditure (in MET-h/week) and the percentage of the samples taken.

The classification and regression tree (CART) [25] was used to predict the values of pregnant women’s PA during COVID-19. In this research, various PPA-related factors served as the categorical factors, and energy expenditure served as the target variable. The splitting criterion was the mean square error, and feature selection was conducted to generate a binary tree. Each CART leaf corresponds to a predictive value equal to the mean energy expenditure. The internal node feature has two functions: “Yes” or “No.” The left branch has the value of “Yes,” and the right branch has the value of “No.” We analyzed the two groups according to the pregnancy experience difference between the pregnant nulliparous and pregnant parous groups. The following are the CART outcomes: among women who were pregnant for the first time, participants aged ≤ 23 years with an annual household income of ≥CNY 100,001–150,000 had the highest predicted value (mean = 10.75 MET-h/week); participants aged > 23 years with a perceived severity score of >3.1 who were in the second trimester of gestation and had a perceived susceptibility score < 2.8 had the lowest predicted value (mean = 3.25 MET-h/week). In the group of women who had been pregnant more than once, those participating with perceived benefits score > 4 had the highest predicted value (mean = 10 MET-h/week); participants aged ≥ 29 with a health motivation score > 4 had the lowest predicted value (mean = 3.4 MET-h/week). Figure 6 and Figure 7 show the categorical factors structure on the predicted value of PPA in pregnant nulliparous and pregnant parous women.

## 4. Discussion

The current research used the HBM to investigate the HBL in nonpregnant and pregnant women, offering novel insight into the relationship between psychosocial and physiological factors and PPA behaviors in 414 Chinese women of reproductive age. The findings reveal most respondents were insufficiently physically active throughout their pregnancy during COVID-19, preventing them from enjoying the benefits of PA, and the HBL in all groups was acceptable. Furthermore, the HBL in the nonpregnant nulliparous group was significantly lower than in pregnant nulliparous and pregnant parous women. HBL was most strongly correlated with exercise energy expenditure in both pregnant nulliparous and pregnant parous women. Overall, the primary hypothesis was validated: the HBL of nonpregnant nulliparous women was lower than that of pregnant nulliparous and pregnant parous women, and PPA was lower than the recommended PA level during COVID-19. Health attitudes and demographic variables were correlated with PPA, which was consistent with the hypothesis.

### 4.1. Overview of Hypothesis Validation Findings

#### 4.1.1. Health-Belief Level (HBL)

This research indicated that the HBL was acceptable among nonpregnant nulliparous and pregnant nulliparous, and pregnant parous women, with perceived severity (only in the pregnant parous group) and health motivation above the median level, whereas perceived susceptibility was poor in the nonpregnant nulliparous group. This indicates that the participants had a favorable attitude toward PPA and exercise during pregnancy, and they believed that they would participate in PPA [26]. However, women lack awareness about PPA; therefore, few women are aware of the dangers of inactivity and the benefits of activity while pregnant [27]. Moreover, nonpregnant nulliparous women had a significantly lower HBL and significantly lower perceived susceptibility, severity, benefits, and barriers scores than pregnant women, which aligns with our hypothesis. They had lower awareness of the hazards associated with inactivity, which may lead to prenatal physical inactivity, harming their health and that of their unborn children or causing illness when they become pregnant [6]. Janakiraman et al. [28] discovered that women who were educated about the advantages and hazards of PA, the dangers of inactivity, and various exercise methods exhibited more favorable attitudes toward PPA. Accordingly, early and systematic education on the benefits and knowledge of PPA based on the current HBL is required for Chinese women, especially young women who have not given birth. In addition, scores for perceived barriers matched the median scores in nonpregnant nulliparous, pregnant nulliparous, and pregnant parous women, suggesting that pregnant women in China may experience or have previously faced barriers to PPA. According to the item score for perceived barriers, the main barriers were laziness, lack of interest, and inconvenience. Perhaps one of the main explanations for this occurrence is that with the growth of the global electronic product industry, the focus is shifting away from sports and toward smartphones, computers, and other electronic devices [29]. Another explanation may be that during the COVID-19 pandemic, individuals were typically required to remain at home which consequently decreased their PA [30].

#### 4.1.2. Physical Activity Status of Pregnant Women

In this study, pregnant women showed the greatest energy expenditure in housekeeping and the lowest in exercise during pregnancy. This indicated that most pregnant women in China did not perform moderate-intensity exercise (e.g., swimming, running, and climbing) during pregnancy. Their total energy expenditure during the COVID-19 pandemic was less than 7.5 MET-h/week recommended by ACOG. Our observations are consistent with the findings of Hori et al. [31] and Ghesquière et al. [32], who reported that pregnant women are usually inactive. After pregnancy, women’s PA progressively declines, a typical occurrence among pregnant women in China and overseas, and this has been particularly apparent during the COVID-19 pandemic [31]. Traditional Chinese society views pregnancy as a delicate time when women should rest and be protected [33]. To prevent miscarriage and minimize pressure from family and friends, Chinese pregnant women prefer to follow traditional advice, such as “Do not leap,” “Do not lift heavy items,” “Do not walk too quickly,” and “Do not walk too much [34].” The above information may partially explain why pregnant Chinese women tend to be inactive and prefer to stroll instead of engaging in more strenuous activities. Meanwhile, social isolation is essential to prevent the spread of the novel coronavirus [35]. Many types of social engagement, including sports, have been halted; people remain pessimistic about these activities because of the current scenario. Pregnant women with weak immune systems are advised to avoid public places as much as possible to minimize the risk of infection [3]. However, the current COVID-19 pandemic necessitates that these individuals engage in enough exercise to boost their resistance to the virus [36], presenting additional obstacles for pregnant women. 

#### 4.1.3. Connection between Health Belief, Demographic Factors, and Prenatal Physical Activity

The correlation analysis demonstrated that HBL, demographic factors, and PPA were linked. Consequently, the HBM may serve as a reliable foundation for investigating variables that influence PPA during pregnancy and further clarifying the relationships between various factors. The HBM dimensions were positively correlated with exercise in pregnant nulliparous and pregnant parous women, which is consistent with the findings of Li Jingfang et al. [37]. This may be because individual perception is inextricably linked to behavior, which underlies the theoretical foundation for knowledge, attitude, belief, and practice (KABP) model and HBM [38]. Meanwhile, HBM and KABP model-based behavioral interventions have been shown to effectively promote smoking cessation, mental illness treatment, and breastfeeding [39]. Accurate and comprehensive perception creates the foundation for individuals to engage in beneficial behavior [38]. People may make erroneous decisions because they have formed false perceptions about a situation [38]. However, perceived barriers, age, BMI, and trimester of pregnancy were negatively correlated with PPA in the pregnant nulliparous group. Fewer physiological variables were linked to PPA in the pregnant parous group. Moreover, higher levels of knowledge and cognition were found in the pregnant parous group, who may encounter fewer obstacles and physical issues [40]. Hence, different groups may perceive the effects of PA differently, depending on their physiological factors and knowledge. Education, income, ethnic group, and geographic location were correlated with exercise- and work-related energy expenditure in pregnant nulliparous and pregnant parous women. Women from higher-income backgrounds who were more educated or who lived in metropolitan areas were more likely to overcome obstacles to engage in regular PPA [41]. Regarding the influence of ethics on PPA, differences were observed between the Han nationality and the minority group, which may be attributed to differences in culture and habits [42].

We also conducted a correlation analysis between demographic factors and HBM dimensions. In the pregnant parous group, there were no physiological variables linked to HBM dimensions. However, age, BMI, and trimester of pregnancy were linked to perceived barriers and health motivation in the pregnant nulliparous group, suggesting that older women had higher BMI, later stage of pregnancy, and more PPA-related obstacles but low health motivation. Pregnant nulliparous women are more likely to avoid PPA because of their weight and stage of pregnancy. They may feel fatigued and be concerned about the possibility of miscarriage [43]. Education, income, and geographic location were linked to HBM dimensions in pregnant nulliparous and pregnant parous women. Women from higher-income backgrounds who had more education or lived in metropolitan areas were more likely to have high HBL. Educational background influences a person’s degree of health perception, and income directly influences their living standard [44]. An individual with a decent income and extensive health knowledge is likely to have greater health cognition and purchase superior health products [44,45].

### 4.2. Classification and Regression Tree

A regression tree is a prediction model that depicts a mapped connection between object attributes and object values. It is a graphical technique that applies probability analysis intuitively and has minimal prediction error [25]. After determining the previously recorded energy expenditure of PA during pregnancy, the object attribute values (demographic and HBM dimensions) were input to build a regression tree, which produced the projected value of the PPA, possibly enabling the creation of intervention strategies. According to the regression-tree results, young women with a high income (annual household income of >CNY 100,001–150,000) and older women with higher perceived susceptibility, severity, and benefits are more likely to participate in PPA during nulliparous pregnancy. The results for the pregnant parous group revealed that perceived benefits had a direct effect on the PPA value and that PPA can be achieved at the recommended level among older pregnant women with greater health motivation, perceived benefits, and severity. This indicates that the impact of perceived benefits was more prominent among pregnant parous women, and they may overcome barriers to participating in PPA if they perceive the advantages, have sufficient health motivation, and are aware of the dangers of inactivity. Additionally, this example demonstrates the value of experience, which may help pregnant women comprehend the advantages of PPA.

The study findings revealed the following major points: (1) more than half (51%) of the participants had an annual income of less than CNY 100,001; (2) the perceived susceptibility, severity, and benefits levels in this survey were not high; (3) pregnant nulliparous women in their second and third trimesters, as well as older pregnant women, were hesitant to engage in PPA; (4) the PPA level was lower than the recommended level during the COVID-19 pandemic; and (5) the item score for media coverage, income, and professional coaching advice in cues to action was high. Additionally, professional prenatal exercise facilities (e.g., pregnancy exercise classes offered by medical institutions and specialized commercial pregnancy exercise clubs) in China are primarily located in developed regions [46]. Consequently, they are costly, and low-income families and those living in underdeveloped areas cannot afford to pay the exorbitant fees [46]. Furthermore, PPA guidelines from a multidisciplinary team of specialists may be hindered by a lack of professional coaches, financial support, and infrastructure during the COVID-19 era. To address the lack of professional institutions, unreasonable prices, safety concerns, etc., female-centered organizations should hold monthly public interest courses for pregnant women at different stages of pregnancy to alleviate the financial strain placed on young people by their families and enhance awareness of the dangers surrounding inactivity and the benefits of activity while pregnant. For instance, women’s associations and communities could increase publicity, universities could invite expert physicians and coaches to offer relevant courses, and a network platform could be established to minimize equipment expenses and ensure safety in the context of COVID-19. For instance, mobile applications (e.g., apps focused on weight management, mental health, nutrition knowledge, counseling medical personnel, and exercise training online) may be beneficial for improving maternal physical and mental health during the pandemic, and affordability represents a major advantage of mobile apps [47,48]. The government may consider providing special activity venues and facilities for pregnant women when planning various community construction projects. Inactivity during pregnancy may be alleviated by providing women with tailored pregnancy education. In addition, evidence-based recommendations on modern prenatal exercise programs should be developed and promoted among pregnant women, exercise and health professionals, and obstetric care providers [49].

### 4.3. Strengths and Limitations

Several advantages and disadvantages of the current research should be taken into account when evaluating the results. First, the sample consisted of nonpregnant and pregnant women who were available and willing to participate from China’s central and western regions. Thus, the results may not be generalizable to pregnant women residing in other parts of China, such as large metropolitan regions, or to ethnic minorities. Second, only self-reported measurements were used to assess PPA levels due to the prohibitive cost and practical difficulty of objectively assessing the PPA levels (e.g., using accelerometers) in such a large group of respondents. Third, this research only gathered cross-sectional data, making it impossible to draw conclusions on longitudinal changes in individual activity levels. Furthermore, prepregnancy PA was not collected from the pregnant participants because of recall bias [50]; consequently, the PA of nonpregnant nulliparous women was not investigated. Longitudinal studies that investigate PA before and during pregnancy in a population-based sample are suggested in the future. Unlike other Chinese studies [37,51,52], this research examined PPA levels in nulliparous and parous pregnancies during the ongoing COVID-19 pandemic. Additionally, HBL was investigated in women of different socioeconomic levels, placing emphasis on reproductive-age women who had not given birth. The correlation between physiological factors and HBL and PPA was verified. The pregnancy HBM can be a vital tool for motivating pregnant women to engage in PA throughout their pregnancy. Prior research has demonstrated that pregnant women generally have a reduced level of PA. However, this study offers much-needed information on changes in the moderators, individual perceptions, and the energy expenditure of PPA performed by pregnant women. We then used a regression tree to estimate the energy expenditure of PA, which may enable the creation of intervention strategies, given that these variables may have varied effects depending on geography, economy, and culture. It is essential to identify the elements that influence women’s exercise programs during pregnancy in various regions and to understand how exercise behavior is connected to individual perception and moderator variables to guide future local policies and practices.

## 5. Conclusions

In this study, demographic characteristics, HBL, and PPA were investigated in Chinese women who were nonpregnant nulliparous, pregnant nulliparous, and pregnant parous. All HBM dimensions had a positive relationship with exercise energy expenditure in both pregnant nulliparous women and pregnant parous women, except for perceived barriers. According to the regression tree, the predicted PPA value would meet the recommended level in young pregnant nulliparous women with high incomes; perceived benefits directly affected the PPA value in the pregnant parous group. These findings may prove useful in establishing population subgroups that require intervention, and they may provide evidence for future recommendations regarding PA during pregnancy. 

## Figures and Tables

**Table 1:** The prenatal physical activity-related health belief model scale.

Dimensions	Item Number	Items	Cronbach’s α
Perceived severity (Belief about how serious a condition and its sequelae are)	1	Prenatal physical inactivity is a severe problem.	0.75
	2	Prenatal physical inactivity can lead to complications, such as obesity, gestational diabetes and gestational hypertension, preeclampsia, and urinary incontinence.	
	3	Prenatal physical inactivity can lead to anxiety and depression.	
	4	Prenatal physical inactivity can lead to post-term pregnancy and cesarean section.	
Perceived susceptibility (Belief about the chances of experiencing a risk or acquiring a condition or disease)	5	Pregnant women do not engage in physical activity.	0.91
	6	Fear of miscarriage can lead to prenatal physical inactivity.	
	7	Some habits can cause prenatal physical inactivity, including disinterest, indolence, and busyness.	
	8	Financial burdens, inadequate equipment, and lack of professional guidance can cause prenatal physical inactivity.	
	9	Recommendations from family members, doctors, and other pregnant women can cause prenatal physical inactivity.	
Health motivation (Awareness of prevention of a risk, condition, or disease)	10	I usually value my health and fetal health.	0.78
	11	I usually take the initiative to acquire prenatal physical activity knowledge.	
Perceived benefits (Belief in the efficacy of the advised action to reduce the risk or seriousness of an impact)	12	Correct and reasonable prenatal physical activity are feasible.	0.92
	13	I can prevent pregnancy complications, such as obesity, gestational diabetes and gestational hypertension, preeclampsia, and urinary incontinence, if I get enough prenatal physical activity.	
	14	I can regulate anxiety and depression if I get enough prenatal physical activity.	
	15	I can promote the health of the fetus if I get enough prenatal physical activity.	
	16	I can reduce adverse pregnancy outcomes if I get enough prenatal physical activity.	
Perceived barriers (Belief about the tangible and psychological costs of the advised action)	17	It is difficult for me to participate in physical activity without being in good physical condition.	0.85
	18	I am lazy and have no interest in pregnancy exercise.	
	19	It is hard for me to get involved in pregnancy exercise if I do not have enough money and belong to a professional maternity organization.	
	20	It is hard for me to get involved in prenatal physical activity without other people supporting me.	
Cues to action (Strategies to activate readiness and promote awareness)	21	Prenatal physical activity information on TV commercials and publication propaganda impact me.	0.80
	22	Prenatal physical activity experiences from family members and friends impact me.	
	23	Views of doctors and coaches on prenatal physical activity impact me.	
Self-efficacy (Confidence in one’s ability to take action)	24	I am willing to participate in prenatal physical activity.	0.81
	25	I can complete the assigned task while participating in prenatal physical activity.	
	26	I can make up my mind to correct my bad habits while participating in prenatal physical activity.	
	27	I can exercise independently during pregnancy.	
Overall			0.91

**Table 2:** Demographic features of the study participants (*n* = 414).

Variable	N (%)
Chinese citizen	414 (100%)
Age (years)	
20–25	161 (38.9%)
26–34	202 (48.9%)
35+	51 (12.2%)
Body mass index	
Underweight	41 (9.8%)
Normal weight	289 (69.9%)
Overweight	59 (14.2%)
Obese	25 (6.1%)
Nationality	
Han	259 (62.5%)
Minority	155 (37.5%)
Annual revenue per capita	
Less than CNY 50,000 per year	108 (26%)
CNY 50,001–100,000 per year	104 (25%)
CNY 100,001–150,000 per year	48 (11.5%)
More than CNY 150,000 per year	128 (31.1%)
Unsure/would rather not say	26 (6.4%)
Highest educational level	
No schooling or primary school	4 (1%)
Secondary/high school	59 (14.2%)
Technical or further educational institution	84 (20.3%)
Bachelor’s degree	208 (50.3%)
Master’s degree	59 (14.2%)
Highest educational level (spouse)	
No schooling or primary school	8 (2%)
Secondary/high school	78 (18.9%)
Technical or further educational institution	76 (18.3%)
Bachelor’s degree	193 (46.6%)
Master’s degree	59 (14.2%)
What is your current number of children?	
None	244 (58.9%)
1 child	122 (29.5%)
2 children	45 (10.9%)
3 or more children	3 (0.7%)
Pregnancy for the first time	
Yes	126 (42.6%)
No	170 (57.4%)
Trimester of gestation	
First trimester	99 (33.4%)
Second trimester	96 (32.4%)
Third trimester	101 (34.2%)
Residential zone	
Urban	141 (34.1%)
Suburban	133 (32.1%)
Rural	140 (33.8%)

## Data Availability

The data that support the findings of this study are available from the corresponding author upon reasonable request.
